# High-Speed Generation of Microbubbles with Constant Cumulative Production in a Glass Capillary Microfluidic Bubble Generator

**DOI:** 10.3390/mi15060752

**Published:** 2024-06-02

**Authors:** Jian Yu, Wei Cheng, Jinchun Ni, Changwu Li, Xinggen Su, Hui Yan, Fubing Bao, Likai Hou

**Affiliations:** 1 Key Laboratory of Measuring & Online Assessment of Energy for Jiangsu Province Market Regulation, Suzhou Institute of Metrology, Suzhou 215128, Chinalicw@szjl.com.cn (C.L.); 2 Zhejiang Provincial Key Laboratory of Flow Measurement Technology, China Jiliang University, Hangzhou 310018, China; 3 Dalian Institute of Metrology Inspection and Testing Co., Ltd., Dalian 116000, China; 4 School of Mechatronics Engineering, Harbin Institute of Technology, Harbin 150001, China

**Keywords:** microbubble, microfluidic, cumulative production, generator

## Abstract

This work reports a simple bubble generator for the high-speed generation of microbubbles with constant cumulative production. To achieve this, a gas–liquid co-flowing microfluidic device with a tiny capillary orifice as small as 5 μm is fabricated to produce monodisperse microbubbles. The diameter of the microbubbles can be adjusted precisely by tuning the input gas pressure and flow rate of the continuous liquid phase. The co-flowing structure ensures the uniformity of the generated microbubbles, and the surfactant in the liquid phase prevents coalescence of the collected microbubbles. The diameter coefficient of variation (CV) of the generated microbubbles can reach a minimum of 1.3%. Additionally, the relationship between microbubble diameter and the gas channel orifice is studied using the low Capillary number (*Ca*) and Weber number (*We*) of the liquid phase. Moreover, by maintaining a consistent gas input pressure, the CV of the cumulative microbubble volume can reach 3.6% regardless of the flow rate of the liquid phase. This method not only facilitates the generation of microbubbles with morphologic stability under variable flow conditions, but also ensures that the cumulative microbubble production over a certain period of time remains constant, which is important for the volume-dominated application of chromatographic analysis and the component analysis of natural gas.

## 1. Introduction

Microbubbles with diameters in microns are characterized by larger specific surface/volume ratios and stronger surface tension, attracting attention from researchers in several fields, such as medical imaging [1,2,3,4,5], drug delivery [6,7,8] and material synthesis [9,10,11,12]. For instance, the reflection, scattering and echo formation of ultrasound waves occur after encountering microbubbles [5], thereby enhancing the contrast of surrounding tissues and imaging effectiveness as ultrasound agents [2,13]. Gao utilized microbubble cavitation and collapse to release an encapsulated drug and dramatically enhance intracellular drug uptake by tumor cells [7]. Lu reported an approach to stabilize carbon dioxide (CO_2_) gas bubbles encapsulated in micron-diameter aqueous drops and obtain thin-shell particles with encapsulated CO_2_ bubbles for core–shell material synthesis [9].

The practicality of microbubbles has driven the development of their fabrication methods [14,15,16,17]. High shear emulsification [18] and pressurized dissolution [19] enable plenty of microbubbles to be generated simply and efficiently. Coaxial electro-hydrodynamic atomization [20,21] offers the advantage of producing smaller-sized microbubbles. However, these methods have difficulty with precisely controlling the diameter/volume and uniformity of microbubbles. Microfluidic technology involves the discretization of fluid flow using liquid and/or gas phases to generate dispersed objects [22,23,24], which has been demonstrated as an efficient method for microbubble preparation [25]. Expected microbubbles can be generated with exquisite control over multiple flows [26]. PDMS microfluidic devices are commonly used to achieve microbubble generation [27,28,29]. Pancholi prepared bubbles with different sizes by controlling the gas–liquid two-phase flow rate ratio and the liquid viscosity in a T-junction device [30]. Chong reported an acoustofluidic method to generate bubbles with different sizes in a flow-focusing configuration. In glass capillary devices [31], monodisperse microbubbles can be generated through the gas–liquid co-flowing mode [32,33], in which the diameter distribution of the generated microbubbles enables a wide range of adjustments because of the processability of tiny capillary orifices less than serval microns in size [34,35,36,37,38]. Zhang reported a co-flow-focusing microbubble generator that produces highly monodisperse microbubbles with diameters ranging from 3.5 to 60 microns [34]. Mei reported an easily-established capillary-based open microfluidic device (COMD) to generate monodisperse bubbles with diameter of 10–300 μm and a generation frequency of 1–1000 Hz [39]. This research has usually focused on the microbubbles’ size [40]. However, limited attention has been given to the research of the cumulative production of microbubbles over a certain period of time and to their uniformity.

**Figure 1:**
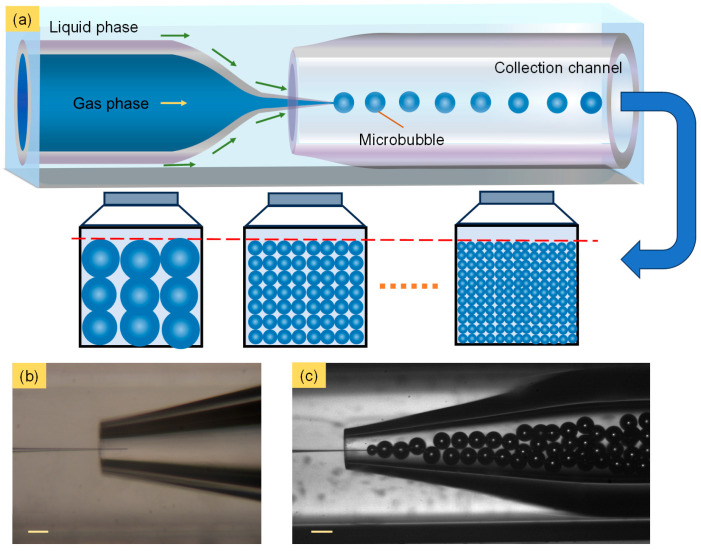
Microfluidic approach for generation of microbubbles with constant cumulative production. (**a**) Schematic illustration of microbubble generator and cumulative production in same period of time. (**b**) Microscopy image of microfluidic glass capillary device. (**c**) Generation of microbubbles. Scale bars are 100 μm.

In this work, we demonstrate that monodisperse microbubbles with constant cumulative production can be generated through a gas–liquid co-flowing glass capillary microfluidic bubble generator, as shown in Figure 1a. The adjustability of the gas channel orifice and the co-flowing structure (Figure 1b) ensure the dimensional controllability and uniformity of microbubbles. Additionally, the avoidance of microbubble coalescence contributed by the surfactant within the generator makes the morphology of microbubbles more stable, as shown in Figure 1c. As a result, uniform microbubbles with specific diameter distributions and coefficients of variation (CVs) were continuously generated under multiple flow conditions. During this process, the relationships between the diameter distribution and control conditions were explored, including the input gas pressure, the liquid phase flow rate and the gas channel orifice. More importantly, a constant cumulative production of microbubbles over a certain period of time was achieved regardless of the liquid phase flow rate, which will contribute to the volume-dominated application of chromatographic analysis and to the component analysis of natural gas.

## 2. Materials and Methods

### 2.1. Materials

Poly vinyl alcohol (PVA, 87–89% hydrolyzed, average Mw = 13,000–23,000) and octadecyltrichlorosilane (OTS) were purchased from Sigma-Aldrich. Nitrogen gas (99.99%) was ordered from Minxing Chemical Co., Ltd. (Shanghai, China). The cylindrical capillary glass tube (ID 0.58 mm, OD 1.03 mm, 1B100-6) was purchased from World Precision Instruments, Inc. (Sarasota, FL, USA). The square capillary glass tube (ID 1.05 mm, OD 1.5 mm, 810-9917) was obtained from Atlantic international technologies, Inc. The surface tension coefficient (σ) was measured using an automatic surface tension meter (BZY-1, Shanghai HengPing Instrument and meter Factory, Shanghai, China). The viscosity (μ) was measured using a Digital Viscometer (NDJ-5S/8S, LICHEN Scientific Instrument Co., Ltd., Shaoxing, China).

### 2.2. Fabrication of the Microbubble Generator 

The microbubble generator was fabricated using a co-flowing microfluidic glass capillary device, as shown in Figure 1a. In detail, two cylindrical capillary glass tubes (1B100-6) were fitted into a square capillary glass tube (810-9917). The two cylindrical capillary glass tubes were tapered by a micropipette puller (P-1000, Sutter Instrument, Novato, CA, USA) and forged by a micro-forge (MF01-M4, China Jiliang University, Hangzhou, China) to 5 μm and 180 μm as the gas channel and the collection channel, respectively. The inner wall of the gas channel was treated with 1 wt% OTS solution to enhance its hydrophobic property. This device is similar to those used in some studies [41,42,43], all belonging to the category of co-flow systems. It is cost-effective and made entirely of glass, providing excellent compatibility with most fluids. The internal position parameters are highly controllable and can be adjusted according to different needs, making it widely applicable.

### 2.3. Generation Process of Microbubbles

In order to stabilize the process of microbubble generation, gas was supplied from a pressurized tank to the gas channel through a polytetrafluoroethylene (PTFE) soft tube. The gas pressure was controlled with a pressure regulating valve (152X-80, GENTEC Shanghai Co., Shanghai, China). The liquid phase (10 wt% PVA solution) was injected into the microbubble generator with a controlled flow rate using a microfluidic controller (PG-MFC series, Precigenome LLC, San Jose, CA, USA). Similar to the co-flowing droplet generation method, microbubbles were formed under the function of shear action of the liquid phase and Plateau–Rayleigh instability at the gas–liquid interface (Video S1). Meanwhile, the process of microbubble generation was captured by a high-speed camera (FASTCAM Mini UX 50, Photron, Tokyo, Japan) with a framerate speed of 5000 fps. Additionally, the generated microbubbles were collected and transferred to a glass culture bottle, where they were left for one minute to stabilize and captured using a COMS camera (Biohd-C12, Fluoca Scientific Co., Ltd., Shanghai, China) to present their morphology and cumulative volume. The PVA surfactant present in the liquid phase played an important role in avoiding the coalescence of collected microbubbles.

## 3. Results and Discussion

### 3.1. Uniformity of Microbubbles at Various Input Gas Pressures

**Figure 2:**
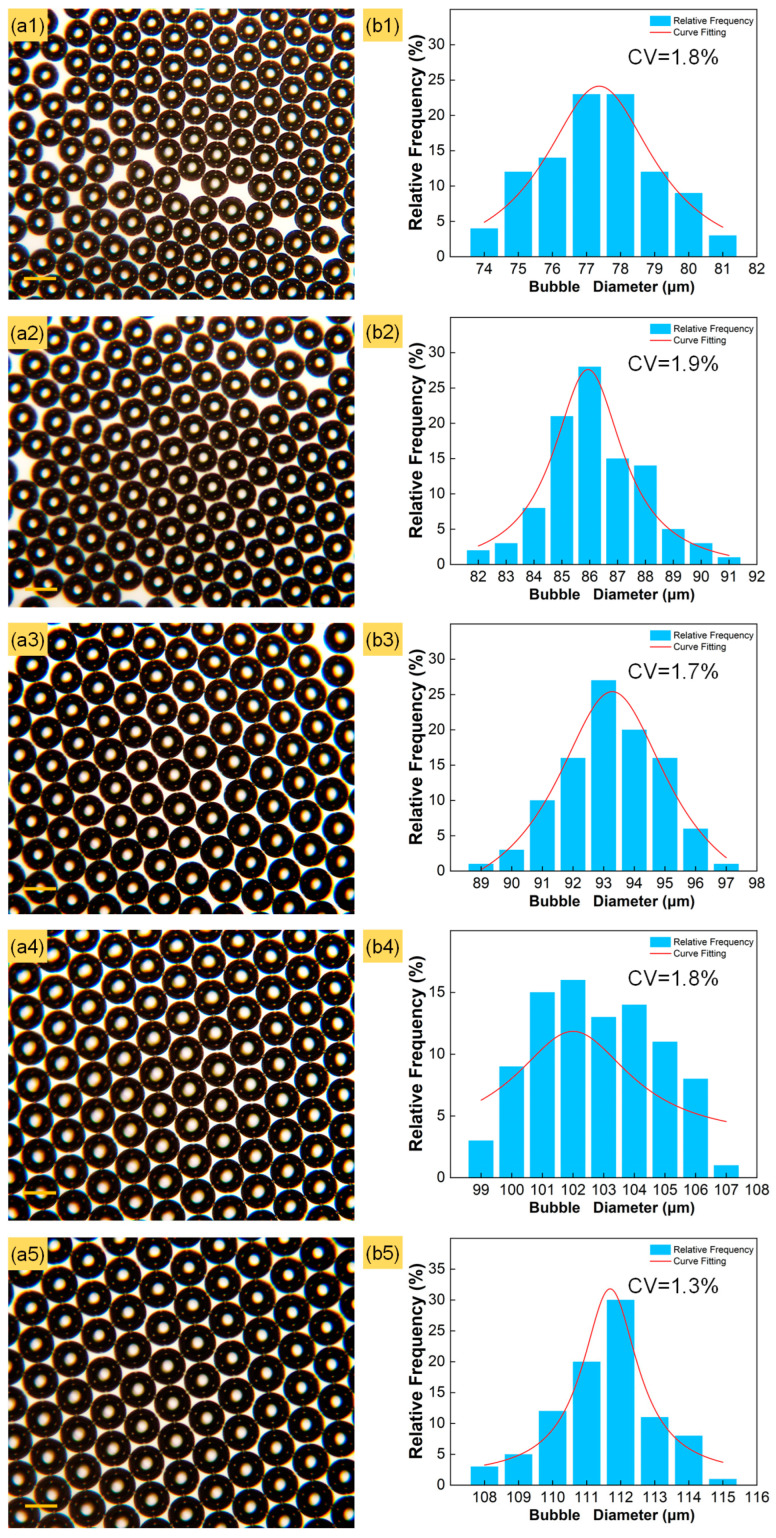
(**a1**–**a5**) Variation in microbubble diameter with different input pressures of 90, 110, 130, 150 and 170 kPa, respectively. Scale bars are 100 μm. (**b1**–**b5**) Diameter distribution of microbubbles corresponding to (**a1**–**a5**).

For a given microbubble generator, the input gas pressure is a critical parameter to control the microbubble morphology. In this experiment, the diameter and distribution of generated microbubbles were calculated at various input gas pressures, as shown in Figure 2, where the gas channel orifice is 5 μm, the input gas pressure is 90 to 170 kPa in 20 kPa increments, and the liquid phase flow rate is maintained at 10 mL/h. In each experiment, 400 microbubbles were selected to analyze their diameters using Image J software (V 1.8.0).

With the increase in the input gas pressure, the diameter of the generated microbubbles increased from 76.87 to 110.02 μm, as shown in Figure 2(a1–a5). This trend is similar to the findings of previous researchers [44,45,46]. A higher input gas pressure results in a larger pressure difference at the tip of the gas channel, leading to a faster gas flow rate and bigger microbubble diameter. In order to demonstrate the uniformity of the generated microbubbles from a data perspective reliably, the distributions and the CVs of their diameters were calculated at different input gas pressures. The CV is calculated as follows:

$$\mathrm{CV}=\frac{D_{\mathrm{st}}}{D_{\mathrm{av}}}\times 100\%$$

D_st_ and D_av_ are the standard deviation of the microbubbles’ diameters and the average microbubble diameter, respectively. 

The CVs under different flow rates of the liquid phase were 1.8%, 1.9%, 1.7%, 1.8% and 1.3%, respectively, as shown in Figure 2(b1–b5). The majority of the generated microbubble diameters were distributed around the median, gradually decreasing within an interval length not exceeding 10 μm at each input gas pressure.

### 3.2. Effect of Different Gas Channel Orifices on Microbubble Diameter

**Figure 3:**
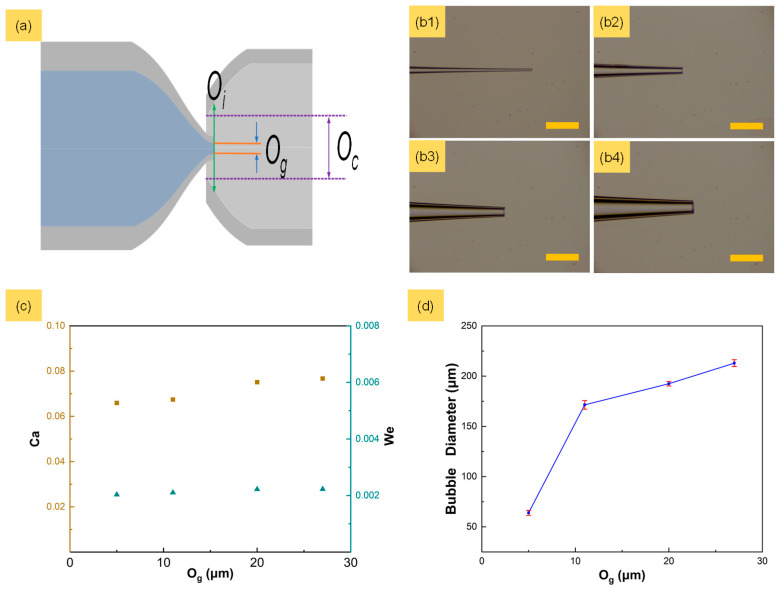
Variation in microbubble diameters with different capillary orifices. (**a**) Diagram illustration of microbubble generator. (**b1**–**b4**) Tapered gas channel with different orifices of 5, 11, 20 and 29 μm, respectively. Scale bars are 100 μm. (**c**) Ca and We at different gas channel orifices. (**d**) Diameter of microbubbles generated at different gas channel orifices.

The gas channel orifice has a significant effect on the distribution of the microbubbles’ diameters. To explore the influence of different orifices on microbubble diameter and ensure the consistency of other parameters, the capillary glass tubes were fixed in the bubble generator according to certain protocols, as shown in Figure 3a. *O_g_* and *O_c_* represent the diameter of the gas channel and the collection channel, respectively. *O_i_* represents the diameter of the cross-section at the junction of the two phases. Four gas channels with orifices (*O_g_*) of 5, 11, 20 and 29 μm were fabricated and fixed to microbubble generators, as shown in Figure 3(b1–b4). Here, *O_c_* and *O_i_* were maintained at 180 μm and 218 μm, respectively. The liquid flow rate and the input gas pressure were maintained at 20 mL/h and 60 kPa, respectively. The Capillary number (*Ca* = *μU*/*σ*) and Weber number (*We* = *ρU^2^l*/*σ*) are both critical parameters for microfluidic devices. Here, *μ* is the viscosity, *σ* is the surface tension coefficient, *U* is the characteristic velocity, *ρ* is the density and *l* is the characteristic length.

In this experiment, different orifices of gas channels resulted in a low *We* of about 0.002 and a *Ca* of about 0.007, as shown in Figure 3c. A larger orifice of the gas channel resulted in a larger microbubble diameter, increasing from 63.94 μm to 212.98 μm, as shown in Figure 3d. The orifice of the gas channel determines the pressure loss of gas and the action of liquid phase viscosity in tapered gas channels under constant flow conditions. As the orifice increased, more time was needed by the liquid phase viscosity to shear the gas column and form microbubbles.

### 3.3. Constancy of Cumulative Microbubble Production at Various Liquid Phase Flow Rates

**Figure 4:**
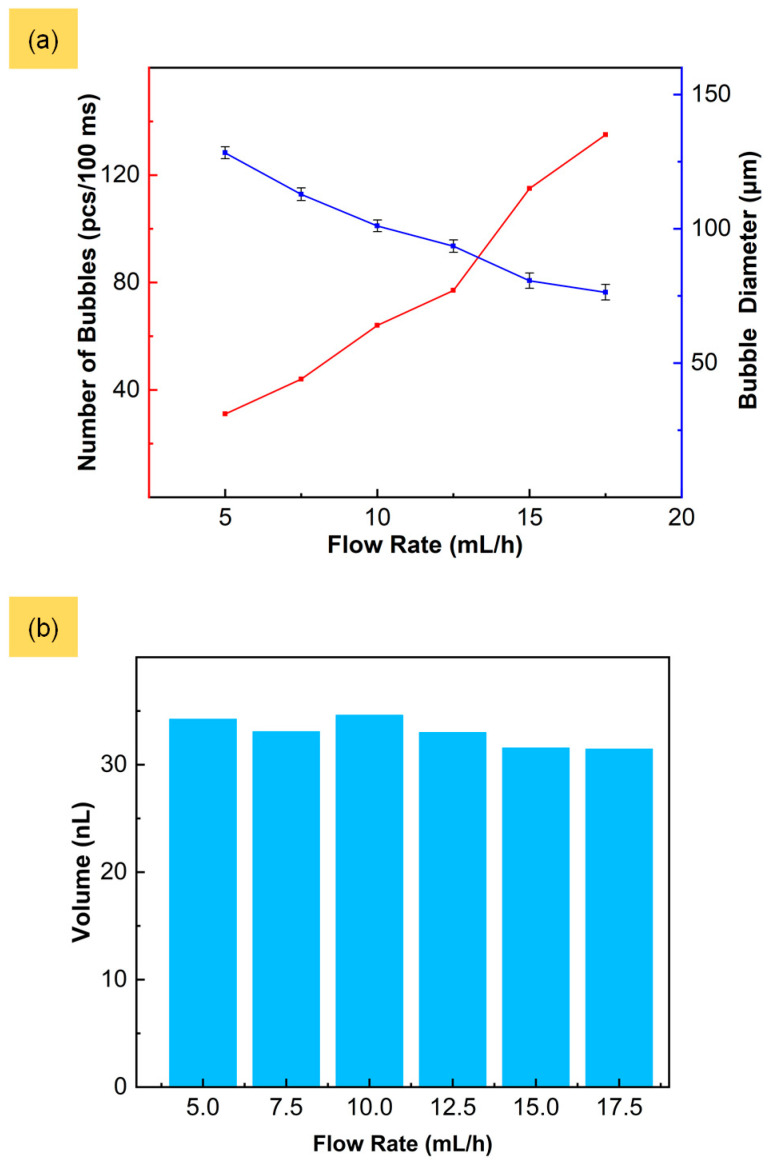
(**a**) Number and diameter of microbubbles with different flow rates of liquid phase. (**b**) Cumulative microbubble production under different flow rates of liquid phase at gas input pressure of 150 kPa for 100 ms.

In conditions of constant input gas pressure, the cumulative microbubble production over a certain period of time remains invariant and unaffected by fluctuations in the flow rate of the liquid phase. This phenomenon can be demonstrated through an examination of both the frequency and diameter of the generated microbubbles at various flow rates of the liquid phase. In this experiment, the input pressure was maintained at 150 kPa, and the flow rate of the liquid phase was increased at 5 mL/h to 17.5 mL/h in 2.5 mL/h increments. Six sets of experiments were performed at each flow rate to obtain the average frequency and diameter of the generated microbubbles. With the increase in the liquid phase flow rate, the microbubbles’ diameters exhibited a downward trend that decreased gradually from 129.11 μm to 76.66 μm, and the frequency of generation increased by about three times, as shown in Figure 4a. 

The viscous shear force and extrusion pressure of the liquid phase increase with a faster liquid phase flow rate, making it easier to overcome the interfacial tension of the two phases. This results in the gradual increase in the shear frequency and the decreased microbubble diameter at the capillary tip. In order to verify the constancy of cumulative microbubble production in such gas–liquid co-flowing microfluidic devices, the total volumes of generated microbubbles per 100 ms at various liquid phase flow rates were calculated, which were 34.23 nL, 33.06 nL, 34.60 nL, 32.98 nL, 31.54 nL and 31.45 nL, respectively, as shown in Figure 4b. The CV of the cumulative microbubble volume can reach 3.6%. The variations in the frequency and diameter of the generated microbubbles did not result in changes in the total volume, indicating that the cumulative microbubble production of the microbubble generator was not influenced by the liquid phase flow rate.

## 4. Conclusions

In this work, we have shown that by using a gas–liquid co-flowing microfluidic device, constant cumulative microbubble production can be achieved with a total volume of around 32.98 nL in 100 ms. The effects of the liquid flow rate, the input gas pressure, and the gas channel orifice on the distribution of the microbubbles’ diameters were explored. Uniform microbubbles can be easily fabricated with diameters between 50 μm and 220 μm by tuning the flow conditions and orifices, and the CV of the microbubbles was less than 1.9%. In addition, despite variations in the flow conditions, the uniformity of the microbubbles’ diameters and morphologic stability were consistently maintained. More importantly, even with variations in the flow rates of the liquid phase, the generated microbubbles maintained a consistent cumulative volume over a certain period time, with the CV reaching 3.6%. This paper demonstrates the robustness and reliability of the device in generating uniform microbubbles across different operating conditions. These results not only contribute to the development of microbubble-related technologies but also hold potential for the volume-dominated application of chromatographic analysis and the component analysis of natural gas. Introducing bubbles into gas chromatography analysis can increase the surface area for gas separation, thereby enhancing the sensitivity and resolution of the analysis. And bubbles can serve as reactors to catalyze or convert reactants in gas-phase reactions, enabling the analysis and detection of gas samples.

## Data Availability

Data are contained within the article.

## Supplementary Materials

The following supporting information can be downloaded at: https://www.mdpi.com/article/10.3390/mi15060752/s1, Video S1: Microbubble generation.
